# Bioengineered 3D microvessels and complementary animal models reveal mechanisms of *Trypanosoma congolense* sequestration

**DOI:** 10.1038/s42003-025-07739-z

**Published:** 2025-02-27

**Authors:** Teresa Porqueddu, Maria Zorrinho-Almeida, Mariana De Niz, Aitor Casas-Sánchez, Viola Introini, Silvia Sanz Sender, Diana Carrasqueira, Luísa M. Figueiredo, Maria Bernabeu, Sara Silva Pereira

**Affiliations:** 1 Instituto Superior Técnico, Universidade de Lisboa, Lisbon, Portugal; 2 Gulbenkian Institute for Molecular Medicine, Lisbon, Portugal; 3 Católica Biomedical Research Centre, Católica Medical School, Universidade Católica Portuguesa, Oeiras, Portugal; 4 Center for Advanced Microscopy and Nikon Imaging Center, Feinberg School of Medicine, Northwestern University, Chicago, USA; 5 Department of Tropical Disease Biology, Liverpool School of Tropical Medicine, Liverpool, UK; 6 EMBL Barcelona, Barcelona, Spain

**Keywords:** Parasite physiology, Parasite immune evasion

## Abstract

In the mammalian host, *Trypanosoma congolense* cytoadheres, or sequesters, to the vascular endothelium. Although sequestration influences clinical outcome, disease severity and organ pathology, its determinants and mediators remain unknown. Challenges such as the variability of animal models, the only-recently developed tools to genetically manipulate the parasite, and the lack of physiologically-relevant in vitro models have hindered progress. Here, we engineered brain and cardiac 3D bovine endothelial microvessel models that mimic the bovine brain microvasculature and the bovine aorta, respectively. By perfusing these models with two *T. congolense* strains, we investigated the roles of flow for parasite sequestration and tropism for different endothelial beds. We discovered that sequestration is dependent on cyclic adenosine monophosphate (cAMP) signalling, closely linked to parasite proliferation, but not associated with parasite transmission to the tsetse fly vector. Finally, by comparing the expression profiles of sequestered and non-sequestered parasites collected from a rodent model, we showed gene expression changes in sequestered parasites, including of surface variant antigens. This work presents a physiologically-relevant platform to study trypanosome interactions with the vasculature and provides a deeper understanding of the molecular and biophysical mechanisms underlying *T. congolense* sequestration.

## Abstract (web summary)

Organ-specific bioengineered microvessels and animal models reveal that *Trypanosoma congolense* sequestration depends on the endothelial cell bed, parasite strain, cyclic AMP signaling and parasite proliferation, but not transmission to the tsetse fly.

## Introduction

T*rypanosoma congolense* is a unicellular, intravascular parasite that causes animal African trypanosomiasis, or nagana, in several mammals, and is particularly pathogenic for livestock and dogs in Africa^1,2^. The parasite replicates in the blood, where it binds to the vascular endothelium, in a process known as sequestration. Whilst most infections result in a chronic disease, a small proportion of animals develop an acute, rapidly fatal illness. In rodent models, acute cerebral disease is determined by increased *T. congolense* sequestration in the brain and is characterized by immune cell recruitment and early death^3^. In other parasitic diseases, such as malaria and babesiosis^4,5^, sequestration also determines clinical course, disease severity, and organ pathology. Sequestration in trypanosomes has recently been suggested to play a role in transmission, since silencing of a gene orthologous to a *T. brucei* negative regulator of differentiation to the insect-transmissible form resulted in reduction of attachment to a plastic substrate and an increase in peripheral parasitaemia in vivo^6^.

Despite its importance for disease pathogenesis, the mechanisms of sequestration, be it molecular, biophysical, or biochemical, remain unknown. Many challenges have precluded the study of sequestration determinants, namely the large phenotypic variability of animal models, the short timespan in the development of acute cerebral trypanosomiasis, and the absence of physiologically-relevant in vitro models that could reproduce the complexity of the parasite-endothelial cell interaction. In cerebral malaria, the development of bioengineered in vitro human 3D brain microvessels^7^ has allowed for the recent development of many advances in severe and cerebral malaria research. In this study, we adapted this microvessel system to the study of trypanosomiasis by engineering two 3D bovine endothelial microvessel models that mimic the bovine brain microvasculature and the aorta. Strains from *T. congolense* that cause acute and chronic trypanosomiasis showed different tropism for cardiac and brain 3D microvessels under different flow mechanical conditions. Using a combination of the microvessels systems, simpler cytoadhesion assays, and mouse and tsetse fly experimental infections, we found that sequestration is dependent on cyclic adenosine monophosphate (cAMP), closely associated to parasite proliferation, but not with transmission. Furthermore, we assessed the *T. congolense* gene expression remodelling that takes place in sequestered parasites.

## Results

**Fig. 1:**
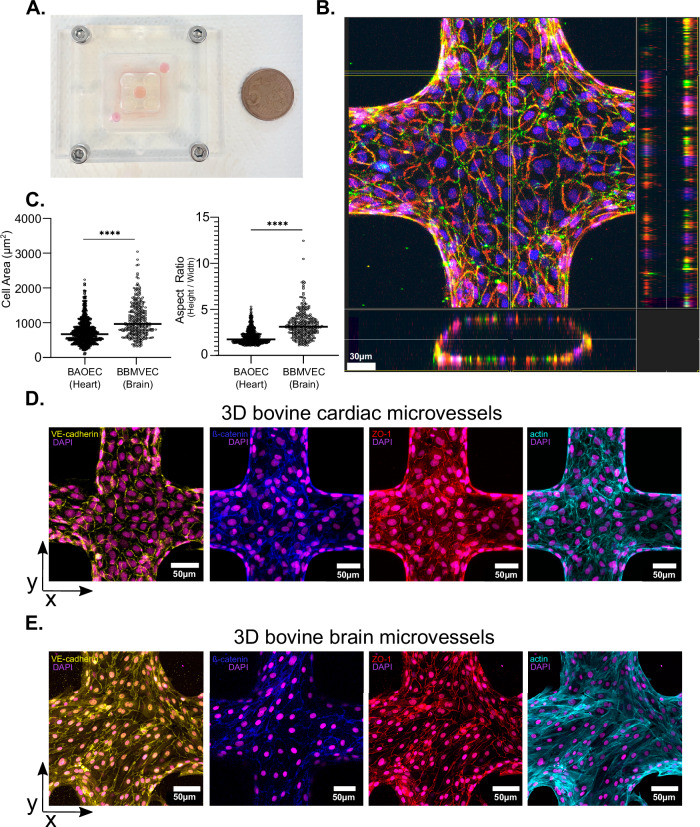
3D bovine microvessels. **A** Representative images of an assembled 3D microvessel device, showing collagen previously injected in between PDMS micro-patterned stamps and PMMA jig, next to a 5 cents coin (21.25 mm). **B** Representative image and orthogonal view of a 3D cardiac microvessel stained for ß-catenin, ZO-1 and DAPI, composed by a single endothelial monolayer displayed in a tubular geometry and empty lumen. Scale bar = 30 µm. **C** Roundness (4 × [Area / (π × major axis^2^)] and aspect ratio (height/width) of bovine brain microvascular endothelial cells (BBMVEC) and bovine aortic endothelial cells (BAOEC) after vessel formation. Unpaired t-test, *p* value < 0.0001. *N *= 3 biologically independent samples. **D** Immunofluorescence z-projections of multiple bovine aortic microvessels showing adherens junction markers (ß-catenin in blue, VE cadherin in yellow), tight junction markers (ZO-1 in red), actin cytoskeleton staining (phalloidin in cyan), and nuclei (4′,6-diamidino-2-phenylindole (DAPI) in magenta). **E** As D, but for bovine brain microvessels. Scale bar = 50 µm.

The use of acute and chronic *T. congolense* rodent models has shed light on the importance of parasite sequestration in vascular pathogenesis and inflammation^3^. Yet, uncovering the determinants of parasite sequestration through the exclusive use of animal models is difficult due to difficulties in disentangling the cellular and biophysical components of the whole organism. To overcome this challenge, we developed a 3D endothelialised bovine microvessel system. The devices consist of a 3D microfluidic network with a pre-defined geometry on a collagen scaffold fabricated by soft lithography and injection moulding (Fig. 1A). The system supports the growth of primary microvascular endothelial cells in lumenised microvessels, perfusable with *T. congolense*, which allows for the study of parasite sequestration under controlled conditions, including flow and endothelial cell type, independently of other host factors.

Our previous results showed that different parasite strains accumulate in various organotypic beds in mouse models, leading to distinct clinical presentations. Whilst *T. congolense* 1/148 parasites accumulate highly in the brain microvasculature, causing acute cerebral trypanosomiasis, IL3000 parasites display tropism for the heart, and cause chronic, wasting disease^3^. To better understand this organ preference in vitro, we developed two microvessel systems: one mimicking the heart vasculature with bovine aortic endothelial cells (BAOEC) and one mimicking the brain with bovine brain microvascular endothelial cells (BBMVEC). Endothelial cell identity of both cell types was assessed by immunostaining on 2D monolayers. Both BBMVEC and BAOEC expressed adherens (VE-cadherin, ß-catenin) and tight junctional markers (ZO-1), as well as the pan-endothelial cell marker, Von Willebrand factor (Supplementary Fig. 1). BBMVEC presented a significantly higher transcriptional expression of the endothelial marker claudin-5 than BAOEC (*p* value = 0.0133, mixed-effects model (REML), Sidak’s multiple comparisons test)), consistent with previous reports in mouse and human cells^8^. Both cell types presented transcriptional expression of endothelial receptors ICAM-1, VCAM-1 and EPCR at similar or higher levels than claudin-5 (Supplementary Fig. 2). We then seeded both endothelial cell types in a 13 × 13 microfluidic branched network of 120 μm microvessels. This geometry recapitulates a large range of flow velocities (42.5-fold range) and generates microvessels analogous to post-capillary venules in terms of surface-to-volume ratio (20 mm^2^/mm^3^). After 3 days in culture, BAOEC and BBMVEC form a 3D monolayer that displays a tubular geometry with an empty lumen (Fig. 1B and Video 1). Immunofluorescence labelling with junctional markers revealed that both cell types align with flow when grown in 3D. However, they are morphologically distinct: BAOEC are smaller and more rounded, whilst BBMVEC are bigger and more elongated with a higher aspect ratio (p < 0.001, unpaired t-test) (Fig. 1C). Nonetheless, they both express adherens (VE-cadherin, ß-catenin) and tight (ZO-1) junctional markers (Fig. 1D and E). The actin cytoskeleton appears to be cortical on BAOEC, while more stress fibres are present on BBMVEC, as previously reported for this bovine cell type^9^. In conclusion, we developed two in vitro microvessel systems mimicking heart and brain vasculature, with expected endothelial and junctional marker expression, to study organ-specific trypanosome sequestration.

### *T. congolense* sequestration to 3D microvessels is dependent on wall shear stress, parasite strain and endothelial cell type

**Fig. 2:**
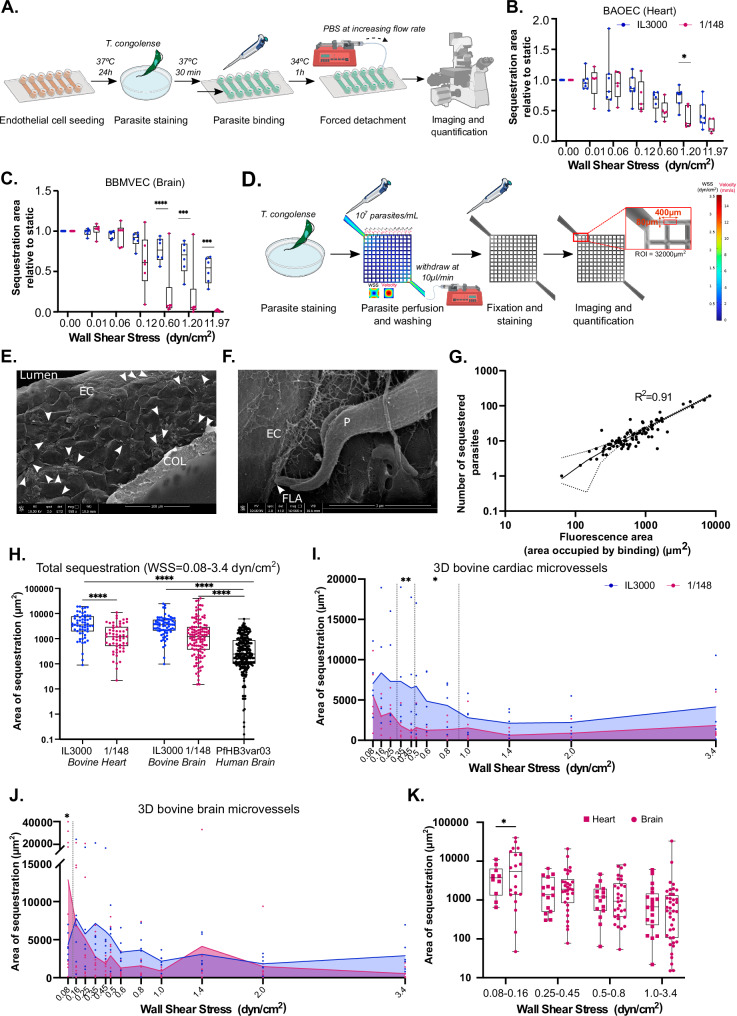
3D bovine microvessels show that *Trypanosoma congolense* sequestration depends on flow. **A** Diagram showing experimental procedures of 2D detachment assay. **B** Number of *T. congolense* parasites (1/148 in pink and IL3000 in blue) remaining sequestered to bovine aortic endothelial cells (BAOEC) after increasing flow rates (median ± range). *N *= 5 for 1/148 and *N *= 7 for IL3000, biologically independent samples. **C** As **B**, but for bovine brain microvascular endothelial cells (BBMVEC). *N *= 6 biologically independent samples. **D** Schematic of 3D microvessel perfusion with *T. congolense*, including estimated wall shear stress and flow velocity simulations (performed with COMSOL Multiphysics) in the 13-by-13 grid geometry at 37 °C, prior to remodelling of the collagen matrix by endothelial cells. **E** Scanning electron micrograph of 3D bovine cardiac microvessel, showing an empty lumen surrounded by a layer of bovine aortic endothelial cells (EC) to which *T. congolense* IL3000 parasites (white arrows) have sequestered, onto a layer of collagen (COL). Bottom bar indicates magnification (958X), working distance (10.6 mm) and scale (100 µm). **F** Zoomed-in section of a scanning electron micrograph of bovine aortic microvessel, showing part of a parasite (P) closely interacting with the endothelial cell (EC) via the flagellum tip (FLA). Bottom bar indicates magnification (50000×), working distance (10.6 mm) and scale (3 µm). **G** Correlation of number of sequestered parasites manually quantified from micrographs and fluorescence area (µm^2^) estimated from Fiji. Pearson’s correlation R^2^ = 0.91, *p* value < 0.0001. **H** Quantification of the total area of sequestration across individual edges of the microvessel device between IL3000 SM and 1/148, in bovine cardiac microvessels and bovine brain microvessels, and with *Plasmodium falciparum* strain HB3var03 in human brain microvessels (median ± range). Stars indicate statistically-significant results (****p value < 0.0001, one-way ANOVA with Tukey’s multiple comparisons test). **I** Area of sequestration occupied in the 3D microvessels at regions exposed to different wall shear stress rates. Line indicates the mean. Statistical analyses were performed for individual wall shear stress values and for binned regions (dotted vertical lines) using a 2-way ANOVA with Sidak’s multiple comparisons test. *p value < 0.05, **pvalue  < 0.01. *N *= 3 biologically independent samples. **J** As in (**I**), but for bovine brain microvessels. *N *= 5 for 1/148 and *N *= 3 for IL3000 biologically independent samples. **K** Quantification of 1/148 sequestration to brain and cardiac microvessels throughout different regions of the device (median ± range) (2-way ANOVA with Sidak’s multiple comparisons test, *p value < 0.05). Some icons of this figure were sourced from Biorender (*created in BioRender. Pereira, S. (2025)* https://BioRender.com/o56s962) or from NIAID NIH BIOART (bioart.niaid.nih.gov/bioart/000503 and bioart.niaid.nih.gov/bioart/000007).

Blood flow velocity and the associated wall shear stress (WSS) varies along the hierarchical vascular bed. In healthy conditions, in the arteriovenous microcirculation, WSS ranges between 5 and 40 dyn/cm^2^ in arterioles and capillaries, and 1 and 5 dyn/cm^2^ in venules^10–12^. However, in pathological conditions, such as when there is vascular obstruction, flow and WSS may reduce^3,10^. To assess the role of WSS and organotypic endothelial cell types in *T. congolense* sequestration, we first determined the mechanical stress that *T. congolense* parasites can withstand. To this end, we used a simpler setup. BAOEC or BBMVEC were seeded into 6-channel µ-slides overnight at maximum confluence. Fluorescently-labelled *T. congolense* parasites were introduced and allowed to cytoadhere for 30 min, followed by perfusion with increasing flow rates to measure parasite binding strength against detachment (Fig. 2A). We observed that both 1/148 and IL3000 parasites remained bound to BAOEC, withstanding high WSS values. In fact, at WSS of 11.97 dyn/cm^2^, 25% ± 11 and 43% ± 21 of 1/148 and IL3000 parasites, respectively, remained sequestered (Fig. 2B). IL3000 presented similar binding kinetics to BBMVEC (51% ± 16 at maximum WSS) (Fig. 2C). Conversely, 1/148 presented a different behaviour on BBMVEC, with parasites being significantly released back to circulation when exposed to WSS forces higher than 0.6 dyn/cm^2^ (*p* value < 0.001, 2-way ANOVA with Tukey’s correction for multiple comparisons). This shows that different parasite strains display heterogeneous binding behaviour to different endothelial beds and suggests that 1/148 presents lower sequestration strength to brain microvessels at physiological WSS.

Having shown that at least a proportion of both *T. congolense* 1/148 and IL3000 can withstand these forces, we tested how WSS determines initial parasite sequestration. When perfused at a constant flow rate of 10 µl/min, endothelial cells in the outer edge of the 13 ×13 grid are exposed to flow velocities that range from 0.4 to 15.2 mm/s, which translates into WSS values between 0.08 and 3.4 dyn/cm^2^. We perfused 3D cardiac or brain microvessels with 1.5 million fluorescently-labelled parasites of either IL3000 or 1/148 strains (Fig. 2D and Video 2). Following a 15-minute perfusion, unbound parasites were washed under flow for 10 min and microvessels were fixed and stained for subsequent microscopical analysis and binding quantification. The edges of the grids were imaged and parasite quantification was performed on 12 regions for which WSS has been previously defined by computational flow simulations^13^, in a region of interest of 3200 µm^2^ per WSS value (Fig. 2D).

Parasites presented widespread cytoadhesion to 3D microvessels by live microscopy. Scanning electron microscopy showed that parasites sequester to the luminal side of the endothelial cells lining the 3D vessels on top of the collagen matrix (Fig. 2E). Within the 3D microvessel, a smooth, empty lumen was surrounded by tightly clustered endothelial cells, to which the parasites sequestered (Fig. 2E). In agreement with previous literature^14^, close interaction between the flagellum and the endothelial cell was observed (Fig. 2F). Several microfilaments coming out of the endothelial cell and surrounding the parasite cell body could also be observed (Fig. 2F). Similar filaments have been previously reported in both *T. congolense* co-cultures with BAOEC^14^ and *T. brucei* interacting with dermal adipocytes^15^.

Having confirmed that parasites sequester to the 3D microvessels, we proceeded with binding quantifications. Since we were using fluorescently-labelled parasites, we calculated the area occupied by parasite binding (obtained from the total fluorescence area) as a proxy for the number of sequestered parasites because these two variables (number of sequestered parasites and fluorescence area) correlate highly to each other (R^2^ = 0.91, Pearson’s correlation, *p* value < 0.001) (Fig. 2G). Overall, IL3000 parasites presented higher sequestration to bovine cardiac microvessels than 1/148 (*p* value < 0.0001, ordinary one-way ANOVA with Tukey’s multiple comparisons test), whilst both strains showed similar binding levels to bovine brain microvessels (Fig. 2H). Remarkably, *T. congolense* sequestration is significantly higher than sequestration of *Plasmodium falciparum* HB3var03 to human 3D brain microvessels, a malaria parasite line associated to severe and cerebral malaria in humans^7,16,17^. *T. congolense* 1/148 presented a 5-fold higher binding, and IL3000 a 15-fold. These results highlight the affinity and extent of binding of *T. congolense* compared to other parasitic disease that affect brain microvessels.

Sequestration of both strains to cardiac microvessels negatively correlated with WSS (Fig. 2I). More specifically, IL3000 presented a high binding level at WSS of 0.5 dyn/cm^2^ and below, then it slowly decreased when it reached a WSS of 1 dyn/cm^2^, after which it plateaued. In contrast, 1/148 displayed lower binding levels than those of IL3000 across all WSS regions, being significantly lower in the range between 0.25 and 0.8 dyn/cm^2^ (*p* value = 0.02 for WSS values between 0.5 and 0.8 and *p* value = 0.001 for WSS values 0.25 to 0.45, 2-way ANOVA with Sidak’s correction for multiple comparisons) (Fig. 2I). These observations agree with findings in mouse models, where *T. congolense* IL3000 shows higher sequestration to the heart microvasculature than 1/148^3^. When exposed to bovine brain microvessels, IL3000 sequestration followed the same binding pattern as cardiac microvessels and reached similar sequestration levels (Fig. 2J). However, 1/148 presented a different sequestration pattern with two independent sequestration peaks. First, sequestration was dramatically high at 0.08 dyn/cm^2^, representing accumulations in 40% of the total vessel area (12867 µm^2^ ± 4483 of sequestration area), which is 3 times what was observed for IL3000 (i.e. 4365 µm^2^ ± 719) (*p* value = 0.03, 2-way ANOVA with Sidak’s correction for multiple comparisons). Then, there was also a non-significant sequestration bump at 1.4 dyn/cm^2^. When comparing 1/148 sequestration across different vascular beds, we observed that 1/148 parasites bound similarly to cardiac and brain microvessels at WSS higher than 0.25 dyn/cm^2^ but presented significantly more binding to brain at pathological WSS levels of less than 0.25 (Fig. 2K) (*p* value = 0.03, 2-way ANOVA with Sidak’s correction for multiple comparisons). Interestingly, in the mouse model of acute cerebral trypanosomiasis, *T. congolense* 1/148 preferentially sequesters in the small capillaries of the brain, often leading to vascular occlusion and vessel blockage^3^, which greatly reduce blood flow and the associated WSS. We conclude that *T. congolense* sequestration is dependent on flow mechanical properties, irrespective of the parasite strain and endothelial cell organotype. Nevertheless, parasite strains display distinct sequestration behaviours, which may be important for the clinical outcome.

### *T. congolense* sequestration can be prevented by interfering with cAMP homeostasis

**Fig. 3:**
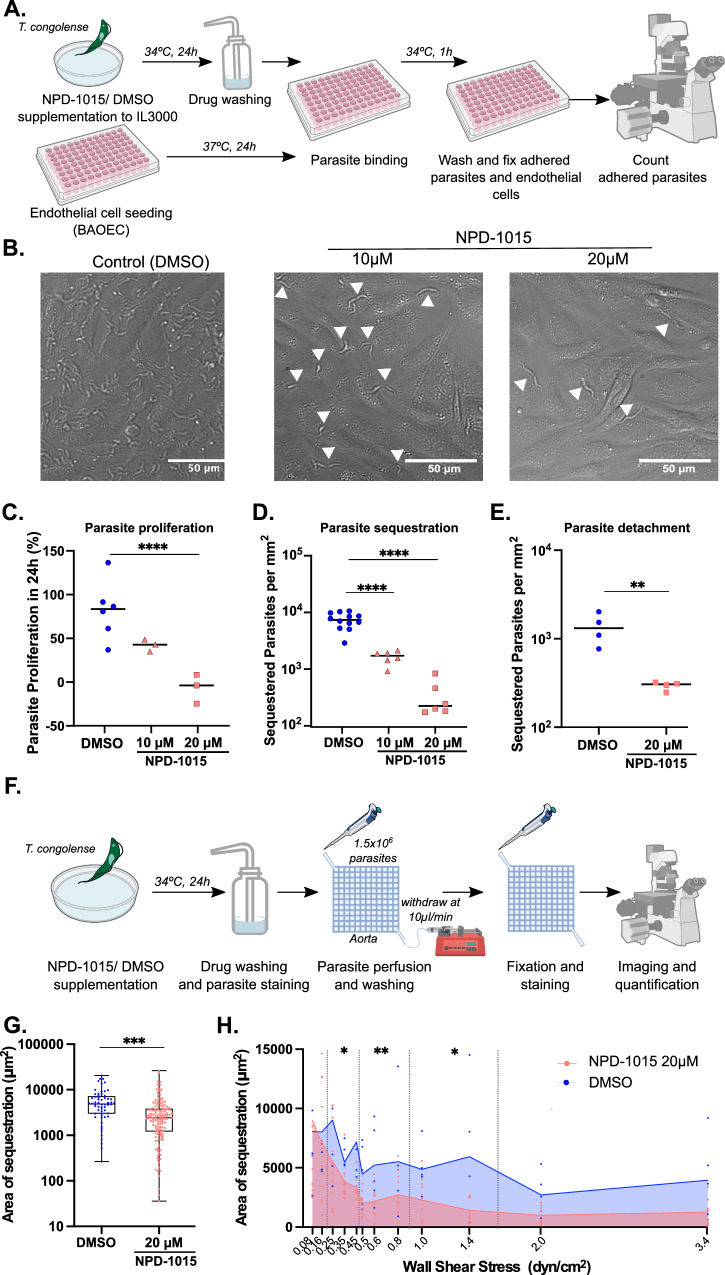
Cyclic AMP homeostasis is essential for *Trypanosoma congolense* sequestration. **A** Diagram showing experimental procedure for testing the effect of NPD-1015 on *T. congolense* sequestration ability. **B** Representative widefield micrographs of *T. congolense* IL3000 parasites sequestered to a bovine aortic endothelial cell (BAOEC) monolayer. Parasites were incubated with DMSO (control), 10 µM or 20 µM of NPD-1015 for 24 h before co-culture with endothelial cells. White arrows indicate parasites. Scale bar = 50 µm. **C** Quantification of parasite proliferation during 24 h of BAOEC co-culture, in the presence or absence of a previous treatment with NPD-1015. *N *= 3 biologically independent samples. **D** Quantification of parasites sequestered to BAOEC with and without previous NPD-1015 treatment (as described in **A** and **B**). *N *= 2 biologically independent samples. **E** Quantification of parasites remaining sequestered to BAOEC after 24-hour treatment with 20 µM NPD-1015. *N *= 4 biologically independent samples. **F** Diagram showing experimental procedure for testing the effect of NPD-1015 on *T. congolense* sequestration to 3D bovine cardiac microvessels. **G** Total area of sequestration between DMSO-treated and NPD-1015-treated parasites across the3D microvessels. Unpaired t-test, ***p value < 0.001. *N *= 3 biologically independent samples. **H** Area of sequestration occupied in the 3D microvessels at regions exposed to different wall shear stress rates. Line indicates the mean. Statistical analyses were performed for individual wall shear stress values and for binned regions (dotted vertical lines) using a mixed-effects analysis with Tukey’s multiple comparisons test. *p value  < 0.05, **p value < 0.01. *N *= 3 biologically independent samples. Some icons of this figure were sourced from Biorender (*created in BioRender. Pereira, S. (2025)* https://BioRender.com/o56s962) or from NIAID NIH BIOART (bioart.niaid.nih.gov/bioart/000503 and bioart.niaid.nih.gov/bioart/000007).

Having established the role of WSS in sequestration, we asked how we could interfere with sequestration in vitro. It has been previously suggested that cAMP phosphodiesterase inhibition results in lower *T. congolense* sequestration^6^. Therefore, we attempted to reproduce that phenotype by treating IL3000 parasites with 10 µM or 20 µM of NPD-1015, an inhibitor of cAMP phosphodiesterases PDEB1 and PDEB2^18^) that interferes with cAMP homeostasis, increasing the cAMP intracellular levels in the parasite and resulting in growth arrest^19^. After 24 h, the drug was removed, and parasites were added to a 2D monolayer of BAOEC and allowed to sequester. Subsequently, we washed unbound parasites and quantified the number of sequestered parasites by microscopy (Fig. 3A and B). Within the timeframe of the experiment, treatment did not affect parasite health: we observed vigorous parasite motility, no abnormal cell debris in the wells, and the percentage of cell death was similar in drug-treated compared to vehicle-treated parasites (10.17% ± 2.16 *vs*. 11.84% ± 2.34, *p* value = 0.4152, unpaired t-test, Supplementary Fig. 3A–C). However, there was a significant growth arrest upon NPD-1015 treatment: whilst untreated parasites proliferated 82% ± 14 over 24 h, parasites treated with 10 µM NPD-1015 grew only 42% ± 4 and those treated with 20 µM NPD-1015 did not proliferate at all (−7% ± 10) (*p* value = 0.0026, one-way ANOVA with Tukey’s correction for multiple comparisons) (Fig. 3C).

We observed that parasite exposed to 10 µM of NPD-1015 presented a 5-fold lower binding (*p* value < 0.0001, one-way ANOVA with Tukey’s correction for multiple comparisons), whilst exposure to 20 µM of NPD-1015 decreased binding levels 20 times (*p* value < 0.0001, one-way ANOVA with Tukey’s correction for multiple comparisons) (Fig. 3B and D). As we washed the drug before adding the parasites to the endothelial cell monolayers, we ensured that any effect observed is parasite-derived. We then asked if NPD-1015 could revert binding of sequestered parasites. Therefore, we co-cultured *T. congolense* IL3000 parasites in a 2D BAOEC monolayer for 24 h. Subsequently, we removed non-sequestered parasites by washing and added 20 µM of NPD-1015 for 24 h longer. We observed a reduction of sequestration, indicating that NPD-1015 induced detachment of parasites. This suggests that increasing intracellular cAMP not only prevents sequestration, but also reverts it (Fig. 3E).

We then used the 3D microvessels system to test whether a similar binding reduction occurred under flow. For that, we incubated parasites with 20 µM NPD-1015 and after 24 h perfused the microvessels with treated parasites (Fig. 3F). Overall, there was a 25% reduction of sequestration (*p* value = 0.03, unpaired t-test) (Fig. 3G), consistent with our previous observation in the 2D assay, but with a lower effect than in EC monolayers. Although NPD-1015 treatment did not affect parasite sequestration dependence on WSS (Fig. 3H), an increase in parasitic cAMP significantly reduced parasite binding in regions exposed to a range of WSS between 0.25 and 1.4 dyn/cm^2^. No differences in binding were observed when the parasite was exposed to a WSS below 0.25 dyn/cm^2^. In conclusion, we showed that interfering with cAMP homeostasis is sufficient to both prevent and revert sequestration, and this effect is more pronounced under specific flow mechanical cues.

### Sequestered parasites proliferate more in the mammalian host and have similar transmission ability to non-sequestered parasites

**Fig. 4:**
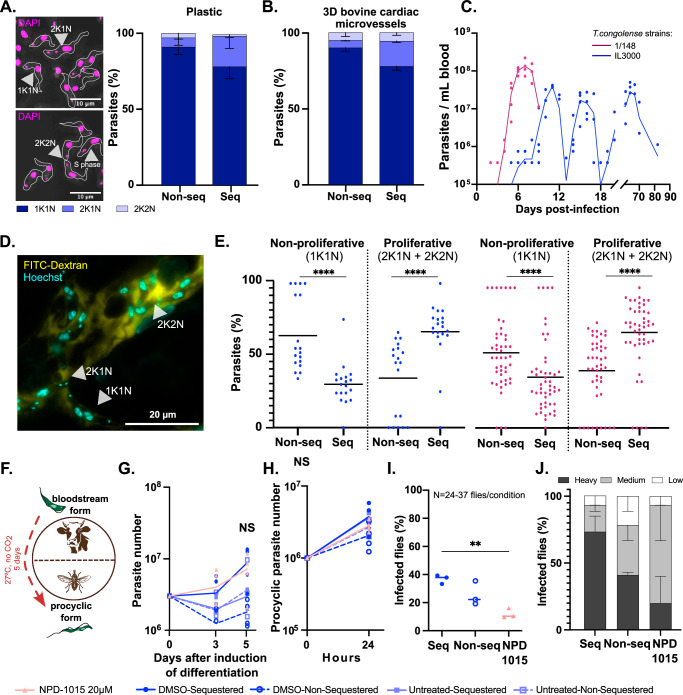
Sequestration is associated with cell cycle but does not affect transmission. **A** Representative image and respective quantification of kinetoplast-nuclei counts of *T. congolense* IL3000 parasites. Nuclei were stained with 4′,6-diamidino-2-phenylindole (DAPI) in magenta. Scale bar = 10 µm. Parasite bodies are delineated in white. *N *= 2 biologically independent samples. **B** Quantification of kinetoplast-nuclei counts of *T. congolense* IL3000 parasites sequestered or non-sequestered to 3D bovine aortic microvessels following 6 h of incubation. *N *= 2 biologically independent samples. **C** Parasitaemia throughout infection with 1/148 and IL3000 parasites estimated by haemocytometry. Line indicates the mean. *N *= 4 biologically independent samples. Adapted from Silva Pereira et al. 2022 (*eLife*). **D** Representative image of kinetoplast-nuclei counts of *T. congolense* 1/148 parasites imaged by intravital microscopy. Nuclei are stained with Hoechst (in cyan) and intravascular space is stained with 70 kDa FITC-Dextran (in yellow). Scale bar = 20 µm. **E** Quantification of kinetoplast-nuclei counts of *T. congolense* 1/148 and IL3000 parasites during mouse infections. Data was collected from 8 major organs (adipose tissue, brain, spleen, liver, kidneys, lungs, heart, and pancreas) throughout the infection course. Stars indicate statistically-significant results (unpaired t-test). ****p value < 0.0001. *N *= 3 biologically independent samples. **F** Simplified representation of the *T. congolense* life cycle, highlighting the differentiation from the mammalian-stage bloodstream form to the insect-stage procyclic form. During in vitro differentiation, *T. congolense* IL3000 SM parasites were incubated at 27 °C without CO_2_ for 5 days to differentiate into procyclic forms. **G** Number of parasites during and after induction of differentiation by temperature and pH alteration, for sequestered and non-sequestered parasites untreated, treated with NPD-1015, or treated with DMSO. Starting parasite populations of 3 million cells. Line indicates the mean. *N *= 4 biologically independent samples. **H** Growth of procyclic parasite population over 24 h starting 5 days after induction of differentiation, when all parasites are procyclic forms. Starting parasite populations of 10^6^ cells. Line indicates the mean. *N *= 4 biologically independent samples. **I** Percentage of tsetse flies successfully infected with sequestered, non-sequestered or NPD-1015-treated *T. congolense* IL3000 SM parasites. Stars indicate statistically-significant results (one-way ANOVA with Tukey’s multiple comparisons test). **p value < 0.01. Line indicates the mean. *N *= 3 biologically independent samples. **J** Tsetse fly midgut infection load at day 10 post-infection. Infections were scored as heavy, mild or low according to the number of parasites observed in dissected midguts. Line indicates the mean. *N *= 3 biologically independent samples.

Next, we hypothesised that sequestration provides an adaptive advantage for *T. congolense*, so that sequestered parasites proliferate faster than non-sequestered. To test that, we examined the cell cycle distribution of sequestered and non-sequestered IL3000 parasites, by quantifying the kinetoplast and nuclei number in each trypanosome cell. Parasites in S phase (i.e. with kinetoplast butterfly-shaped) were considered as 2K1N. First, we assessed it in parasites attached or not to a plastic substrate and observed that the population of sequestered parasites contained a higher proportion of proliferating parasites, which typically can be distinguished by having two kinetoplasts and one nucleus, relative to non-sequestered parasites (2K1N, 20% ± 8 *vs*. 6% ± 5, mean ± sd, *p* value < 0.0001, one-way ANOVA with Sidak’s correction for multiple comparisons) (Fig. 4A), suggesting that sequestered parasites divide more frequently than non-sequestered parasites even without the presence of endothelial cells. We did not observe statistically significant differences in the number of mitotic parasites (2K2N configuration). Subsequently, we assessed it within 3D microvessels. This required perfusing the microvessels with Hoechst-stained parasites for DNA visualisation, incubating them for 6 h so that any change in cell cycle profile could be detectable, washing the non-sequestered parasites and any debris, fixing the devices and their cells, and imaging. For the non-sequestered group, we collected those parasites that did not attach to the microvessels and flowed through the outlet of the device. Again, sequestered parasites displayed 2K1N configuration more often than non-sequestered relative to non-sequestered population, (17% ± 1 *vs*. 5% ± 1, mean ± sd, *p* value = 0.0216, one-way ANOVA with Sidak’s correction for multiple comparisons) (Fig. 4B). This shows that the shift in cell cycle profile between sequestered and non-sequestered parasites is noticeable at least from 6 h post-attachment. Then, to test whether this would apply in vivo, we used intravital microscopy data previously collected from mice infected with either *T. congolense* 1/148 or IL3000^3^. In 1/148 infections, we analysed video recordings from 8 major organs (i.e. adipose tissue, brain, heart, liver, lungs, kidneys, spleen) at days 1 to 6 post-infection, corresponding to the timepoint after which infected animals start developing acute cerebral trypanosomiasis. In IL3000 infections, we analysed data from the same organs, but at the first peak of parasitaemia, the interval between the first and the second peaks of parasitaemia, where peripheral parasitaemia is barely detected, and the second peak of parasitaemia (Fig. 4C). Before image acquisition, Hoechst and FITC-dextran were injected intravenously into the mice, to allow detection of intravascular parasites and their DNA using intravital microscopy (Fig. 4D). We differentiated between sequestered and non-sequestered parasites based on their displacement during the video as previously described^3^. We observed that, overall, sequestered parasites were more often found replicating and dividing (i.e. 2K1N or 2K2N) than non-sequestered parasites (*p* value < 0.0001, one-way ANOVA with Tukey’s correction for multiple comparisons) in both 1/148 and IL3000 infections (Fig. 4E). This shows that sequestered parasites divide more than non-sequestered parasites, irrespective of the parasite strain.

Finally, we asked whether the tissue microenvironment might affect parasite division (Supplementary Fig. 4). In both IL3000 and 1/148 mouse infections, we observed an enrichment of dividing parasites (2K1N and 2K2N) in all organs, regardless of the parasite strain, except on the lung and kidney for 1/148. Therefore, we conclude that the association between sequestration and *T. congolense* cell division is neither strain- nor endothelial cell organotype-dependent.

Given our results suggesting that sequestration facilitates parasite proliferation (or vice-versa) and recent work suggesting that non-sequestered parasites may be growth-arrested, insect-transmissible forms^6^, we tested the ability of sequestered and non-sequestered parasites to differentiate into procyclic (insect) forms (Fig. 4F). We separated in vitro sequestered from non-sequestered parasites, incubated them in differentiation trypanosome media (DTM), at 27 °C, without CO_2_, and followed parasite differentiation and growth for 5 days (Fig. 4F). We observed that both sequestered and non-sequestered parasites could successfully differentiate into procyclic forms with similar dynamics, reaching similar parasite number within 5 days (Fig. 4G). Procyclic parasites were identified by their morphology (pointy and elongated cells, with the flagellum starting from the mid body) and motility (not sequestering, fast swimmers). We also quantified their proliferation rate over 24 h after procyclic differentiation and did not observe any difference (Fig. 4H). Since cell cycle arrest precedes procyclic differentiation in the related organism *T. brucei*^20^, we forced *T. congolense* cell cycle arrest with the administration of NPD-1015 24 h before induction of differentiation. Again, we did not observe any difference in the ability of each parasite population to differentiate into procyclic forms (Fig. 4G) or of differentiated procyclic forms to grow (Fig. 4H). Finally, we assessed the ability of sequestered, non-sequestered, and NPD-1015-treated bloodstream form parasites to infect tsetse flies. As *T. brucei* PDEB1 gene deletion was shown to disrupt social motility^21^ and pH taxis^22^ of procyclic parasites, we thoroughly washed the parasites before feeding them to the flies, to remove any traces of NPD-1015. Furthermore, we fed the tsetse flies with a low inoculum (10^5^ parasites/ mL blood) to increase the probability of observing differences in fly-infectivity. The lower the inoculum, the higher the proportion of parasites that must be competent to achieve infection. In contrast, with a higher inoculum, even a small proportion of fly-infective trypanosomes could represent enough parasites to saturate fly infection rates and mask potential differences between groups. We observed that sequestered and non-sequestered parasites infected similar proportions of flies (37% ± 3 and 26% ± 9, respectively), whereas NPD-1015-treated parasites infected significantly fewer flies (12% ± 4) (*p* value = 0.0043, one-way ANOVA with Tukey’s correction for multiple comparisons) (Fig. 4I). Moreover, despite similar overall infection rates between sequestered and non-sequestered parasites, we observed that the former resulted in heavier infections (higher parasite load in the midguts) than both non-sequestered and NPD-1015-treated parasites (73% ± 20 *vs*. 41% ± 4 *vs*. 20% ± 35 (mean ± sd); *q* value = 0.0002, < 0.0001, = 0.021, respectively, 2-way ANOVA with Benjamini, Krieger and Yekutieli method correction for false discovery rate) (Fig. 4J).

In summary, our data suggest that sequestration is associated to higher proliferation rates in the mammalian host and heavier infections in the vector, which might affect transmission potential, even though both sequestered and non-sequestered parasites are fly-transmissible.

#### *T. congolense* sequestered parasites show distinct transcriptomes to non-sequestered

The striking differences in cell cycle stage between sequestered and non-sequestered parasites both in vitro and in vivo, suggest that these two parasite forms are intrinsically distinct. Therefore, we compared their gene expression profiles during acute cerebral trypanosomiasis in vivo. We infected C57BL/6 J mice with 2000 *T. congolense* 1/148 parasites^23^ and, at the first peak of parasitaemia (day 6 post-infection), we collected systemic blood and three organs: the brain, the adipose tissue, and the kidney. From these samples, we extracted total RNA and performed multiplexed trypanosome targeted RNA sequencing based on the spliced-leader^24^ enrichment (SL-seq)^25,26^. These organs were chosen because the parasite population in their vasculature is predominantly in its sequestered form^3^. Therefore, we obtained transcriptomes of non-sequestered parasites from systemic blood samples, whereas transcriptomes enriched for sequestered parasites were obtained from the tissues.

**Fig. 5:**
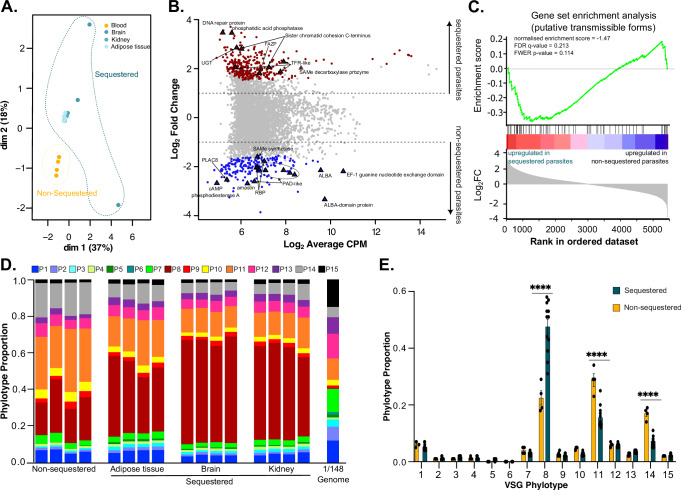
Sequestered parasites display differential transcriptomes and changes in VSG expression. **A** Multidimensional scaling plot showing distribution of sequestered and non-sequestered parasite samples and the tissues they derive from. **B** MA plot showing differentially expressed genes (Log_2_ Fold Change) and their abundance (Log_2_ counts per million transcripts (CPM)). Genes mentioned in the text are labelled and represented by a triangle. **C** Gene set enrichment analysis^65,66^ using genes upregulated upon silencing of gene TcoREG9.1 as query. Genes were pre-ranked by their log_2_ fold change value upon sequestration. Gene distribution is shown by black arrows along the full ranked transcriptome. Green line indicates distribution of enrichment scores. **D** Expressed variant antigen profiles of sequestered and non-sequestered parasites per mouse and per organ of origin, compared to the *T. congolense* 1/148 genomic variant antigen profile. Phylotypes are colour-coded according to key. **E** Expressed variant antigen profiles of sequestered and non-sequestered parasites. ****pvalue < 0.0001, unpaired *t*-test. Bars are colour-coded according to key. *N *= 4 biologically independent samples.

First, we removed the sequencing reads that mapped to the mouse genome. Then, we mapped the remaining reads to the annotated *T. congolense* IL3000 genome^27^ (Supplementary Data 1), given that genome homology analysis shows that IL3000 and 1/148 strains have 94.8% ± 2.8 nucleotide sequence identity (Supplementary Fig. 5)^28^. We compared the transcriptomes from parasites of each tissue and blood to see if we were able to detect significant tissue-specific differences. We observed that the transcriptomes of non-sequestered parasites (from the systemic blood) clustered together (Fig. 5A). Correlation analysis further showed that transcriptomes of sequestered parasites were more different from non-sequestered parasites, than sequestered parasites collected from different tissues (*R*^2^ = 0.58-0.61 *vs*. 0.91-0.96, Pearson’s correlation). Therefore, in subsequent analyses, we compared the transcriptomes of sequestered parasites irrespective of the tissue they derived from to the group of non-sequestered parasites. We detected 523 differentially expressed genes, of which 323 were upregulated in sequestered parasites (Fig. 5B). Upregulated genes included phosphatidic acid phosphatase, DNA repair protein, sister chromatid cohesion C-terminus, UDP-Gal/UDP-GlcNAc-dependent glycosyltransferase (UGT), transferrin receptor-like proteins (both Fam14 and 15) and the orthologue to flagellum attachment zone protein (FAZP). Downregulated genes included those encoding for ALBA and other RNA-binding proteins, PAD-like genes (protein associated with differentiation), amastin, cAMP phosphodiesterase A, and PLAC8 family (Fig. 5B).

We asked if genes previously identified as upregulated in *T. congolense* parasites upon silencing of a negative regulator of differentiation to insect-transmissible forms (TcoREG9.1)^6^, and that therefore could be assumed to be characteristic of insect-transmissible parasites, were enriched within our dataset. We did not find compelling evidence of enrichment (Fig. 5C). Based on what we know from *T. brucei*, at the peak of infection, the population of parasites is expected to contain more insect-transmissible forms than in the ascending phase of infection^29^. Therefore, we also tested a gene set comprising genes upregulated in the first peak infection compared to the ascending phase of infection^29^, but also did not find any evidence of enrichment. Together, these results corroborate our previous observation that *T. congolense* sequestration (or their lack of) is not associated with transmission ability.

Given that sequestration is a physical interaction between the parasite and the endothelial cell and considering that the *T. congolense* cell surface is tightly packed with the major antigen, variant surface glycoprotein (VSG), we specifically looked for changes in their expression. VSGs cannot be accurately characterised using standard differential expression tools, so we used the software VAPPER^30^ to profile them in sequestered and non-sequestered parasites. *T. congolense* VSGs cluster into 15 phylogenetically-distinct lineages (or phylotypes), between which genetic recombination is rare^31^. We found genes from all phylotypes being expressed at the mRNA level, consistent with previous observations in metacyclic and bloodstream forms (Fig. 5D)^31,32^. However, we found that genes belonging to VSG phylotype 8 were predominantly expressed in sequestered parasites, irrespective of the mouse organ, whereas genes from phylotypes 11 and 14 were more abundant in non-sequestered parasites (*p* value < 0.0001, 2-way ANOVA with Sidak’s multiple comparisons test) (Fig. 5D and E). These results suggest functional differentiation amongst the VSG repertoire and a role of phylotype 8 genes in sequestration.

Our results support the conclusions that sequestered and non-sequestered *T. congolense* bloodstream forms present different transcriptomes, and that sequestration might be directly linked to VSG expression.

## Discussion

Sequestration is emerging as an essential process of *T. congolense* interaction with the mammalian host, although its mechanisms remain unknown. In this work, we have significantly improved our understanding of trypanosome sequestration through the development of physiologically-relevant in vitro systems and their use in combination with in vivo animal models. We have discovered the importance of flow mechanical cues as an important determinant of sequestration, revealed that cAMP intracellular levels modulate sequestration, found a link between sequestration and cell cycle that is independent of transmission ability, and characterised the gene expression profiles of sequestered parasites.

Novel bioengineering tools are gaining relevance in the infection biology field. One of their main advantages is the possibility to generate animal species-specific tissues in vitro to study zoonoses or veterinary infections. To the best of our knowledge, we have generated for the first time a non-human microvessel model and showed their potential to study animal pathogens. By modelling bovine small arterioles or postcapillary venules, this system mimics the natural host’s endothelium and the preferred environment for trypanosome sequestration. One limitation of this study is the use of primary aortic endothelial cells in a cardiac model of small coronary arterioles or postcapillary venules. The microvessel diameter of 120 µm is considerably smaller than a bovine aorta, which might have implications in BAOEC differentiation and/or organisation. In the future, adapting the current model to primary cardiac microvascular endothelial cells could be considered. Despite low throughput and technical complexity of microfabrication, our versatile bioengineered method supports the generation of organ-specific vasculature and approximates in vitro settings to natural conditions, by modelling a wide range of physiological WSS and flow velocities within a single device. Future studies could explore these models as platforms to test sequestration mediators or study endothelial cell responses to trypanosomes. In the malaria field, similar approaches have led to a better understanding of cerebral malaria, such as the identification of polyclonal^13^ or monoclonal antibodies^33^ that inhibit parasite binding, or the discovery of new mechanisms of brain microvessel dysfunction^34,35^. In this study, we have exploited them to reveal the biophysical and molecular determinants of *T. congolense* sequestration as well as suggest a link between parasite binding and proliferation. Furthermore, microvessels might help us to characterise mechanisms of vascular transmigration of tissue-invading related trypanosome species, such as *T. brucei*^15,36–39^. In this study, we restricted perfusion experiments at conditions of low and homogeneous viscosity (Newtonian fluid). This model can be used in the future to test how viscosity and potentially the presence of blood cellular components affect sequestration, or assess the role of haematocrit levels in sequestration and/or extracellular matrix invasion.

*T. congolense* can withstand high WSS both in 2D and 3D vascular models, corroborating previous observations of parasites sequestering in large arteries of the mouse^3^, where WSS is approximately around 10 dyn/cm^2^. However, based on our microvessels models, sequestration gradually increases as WSS decreases. Indeed, maximum sequestration is achieved in pathologically low WSS values, when combining *T. congolense* 1/148 and brain endothelium. This is reminiscent of acute cerebral trypanosomiasis, where brain pathology is associated with high parasite accumulation in the microvasculature and vascular occlusion, which ultimately culminates in ischaemic or haemorrhagic stroke-like events^3^. Future studies could assess if selection and expansion of *T. congolense* IL3000 parasites to brain endothelium could result in increased affinity to this cell type and potentially increased disease severity in mice. A similar panning strategy has proven to be useful for the selection and discovery of *P. falciparum* variants and *var* genes and endothelial cell receptors associated with severe disease^16,40^.

The subtle but significant differences in the effect of WSS in sequestration between parasite strains and host endothelial cell types suggest that there may be more than one host ligand and/or parasite receptor of sequestration. Indeed, research in *P. falciparum*, has revealed that binding heterogeneity arises from the combination of different host receptors and parasite ligands from the PfEMP1 family^7^. Here, we found several surface-expressed proteins upregulated in sequestered parasites, including VSGs, invariant surface glycoproteins, and flagellum attachment zone proteins. Furthermore, we found VSG phylotype 8, which has been previously found to be abundant in metacyclic and bloodstream form parasites of different strains, despite is small prevalence in the genome ( ~ 12 genes in a total *VSG* gene pool of >1700)^31,32^, to be significantly more abundant in sequestered parasites. Although our data is limited in sample size (4 infected mice) and restricted to a single parasite strain, future research could investigate further if these candidates are sequestration mediators. Moreover, although we did not find significant differences in the expression of ICAM1, VCAM1, and EPCR in aortic and brain endothelial cells under basal conditions, the presence of *T. congolense* in the vasculature or the resulting inflammatory environment might lead to differential protein expression between endothelial cell beds and explain variations in parasite binding between tissues.

Previous studies suggested that sequestration to live, but not fixed, bovine aortic endothelial cell monolayers increased *T. congolense* proliferation^41^. Here, we corroborated these results by showing that, in vivo, *T. congolense* parasites divide more when sequestered. These data suggest that sequestration might bring a metabolic advantage for parasites, i.e. the physical contact between the parasite and the endothelial cell may facilitate hijacking of host cellular functions and/or nutrients. However, here we uncovered an additional layer of complexity: faster cell division is observed even when the parasite is attached to a plastic substrate although at a lower rate than in the presence of endothelial cells. This indicates that the link between sequestration and proliferation is partly intrinsic to the parasite and not just a consequence of a potential metabolic benefit.

It has been previously suggested that sequestered and non-sequestered parasites could be two distinct life forms: the first adapted for proliferation in the mammalian host (analogous to the *T. brucei* slender form), and the latter adapted for fly transmissibility (analogous to the *T. brucei* stumpy form)^6^. However, our results suggest that both sequestered and non-sequestered parasites can differentiate to the insect stage in vitro and successfully infect tsetse flies in similar timeframes. This could be because the number of parasites in G0/G1 (1K1N) present in the sequestered parasite population are sufficient to establish a successful procyclic form population, masking the phenotype of the remaining proliferating population. However, in these conditions, we would have expected faster differentiation of non-sequestered populations (as the number of parasites in G0/G1 is larger) and of NPD-1015-treated parasites (because this drug induces growth arrest and detachment). Instead, our results strongly suggest otherwise: NPD-1015-treated parasites infect flies at lower rates (perhaps because cAMP is important for successful infection, as reported for *T. brucei*^42^), and sequestered parasites result in heavier fly infections, which might result in higher transmission risk if transposed to heavier mouthpart infections. An alternative is that cell-cycle arrested forms are not necessary for fly transmission, which would explain why differentiation to procyclic forms is not more efficient if the starting population has more cells in G0/G1. A similar hypothesis, suggesting that proliferative *T. brucei* parasites are capable of infecting flies, has been recently proposed^43^.

While it remains unclear why sequestration promotes cell division, or vice-versa, cAMP homeostasis appears central to this question. When we inhibited cAMP phosphodiesterases with NPD-1015, we observed growth arrest, but also a drastic decrease in sequestration. NPD-1015 treatment not only prevented attachment to endothelial cells, but also caused detachment of already sequestered cells. It has been thoroughly described that cAMP phosphodiesterase inhibition induces growth arrest in mammalian cells^44,45^, and alters endothelial cell permeability and barrier properties^46–49^, which may result in altered expression of surface molecules. As we washed the drug away before adding the parasites to the endothelial cells, we show that NPD-1015 prevents *T. congolense* sequestration independently of endothelial cells, but parasite detachment could be partly result from changes in endothelial cell biology. Although, previously, it was shown that a similar inhibitor was able to prevent adhesion of *Crithidia fasciculata*^50^, here we observed detachment of already attached cells. Therefore, whilst cAMP signalling might play an evolutionarily-conserved role in kinetoplastid parasite attachment, the specific effects in sequestration seem distinct in *T. congolense* bloodstream forms.

To conclude, we present a new experimental model for assessing trypanosome interactions with the vascular endothelium and add new insights into the roles and characteristics of *T. congolense* sequestration. Our work lays the ground for additional mechanistic examinations of sequestration, including receptor-ligand discovery.

## Methods

### Animal Experiments

This study was conducted in accordance with EU regulations and ethical approval was obtained from the Animal Ethics Committee of Instituto de Medicina Molecular (IMM) (AWB_2021_11_LF_TrypColonization), the Animal Ethics Committee of the European Molecular Biology Laboratory, and the Animal Ethics Committee of Instituto Gulbenkian de Ciência (IGC) (A003.2023). We have complied with all relevant ethical regulations for animal use. Infections were performed either at the rodent facility at the Parc de Recerca Biomedica de Barcelona (PRBB), where EMBL Barcelona is located, at IMM’s rodent facility, or at IGC’s mouse facility, in 6–10 weeks old, wild-type, male C57BL/6 J mice. Mice were infected by intraperitoneal injection of 10^6^ [*T. congolense* savannah 1/148 (MBOI/NG/60/1-148)^23^. Blood for parasite isolation for perfusion of 3D microvessels was obtained by cardiac puncture. Mice were sacrificed by CO_2_ narcosis.

### Cell culture

Bovine brain microvascular endothelial cells (BBMVEC) (Cell Applications #B840-05), Bovine aortic endothelial cells (BAOEC) (Cell Applications #B304-05) and Human brain microvascular endothelial cells (HBMEC) (Cell Systems #ACBRI 376) (used for Human brain microvessels) were cultured as per supplier’s instructions respectively in bovine brain endothelial cell growth media (Cell Applications, USA), bovine endothelial cell growth media (Cell Applications, USA), or in complete endothelial growth media-2MV (Lonza) containing 5% foetal bovine serum. *T. congolense* savannah 1/148^23^ (MBOI/NG/60/1-148) were expanded in 7–10 weeks-old, male C57BL/6 J mice (Charles River, France), harvested from blood by cardiac puncture and purified by anion exchange chromatography. *T. congolense* savannah IL3000 SM^51^ parasites were cultured in HMI-93 medium supplemented with 10% goat serum on 10mm-diameter Petri dishes, at 34 °C, until 80–100% confluency. When necessary, parasite cells were stained with fluorescent dye 5(6)- Carboxyfluorescein diacetate succinimidyl ester (CFDA-SE), at a 1:1000 dilution, as per manufacturer’s instructions. *Plasmodium falciparum* clone HB3 was selected for expression of PfEMP1 variants HB3VAR03 as previously described^52^, and cultured in human O^+^ erythrocytes in RPMI-1640 medium containing 25 mM HEPES, 4 mM L-glutamine, 0.04 mM hypoxanthine, 5 mM glucose, and 10% human type B+ serum at 37 °C and 90% N_2_, 5% CO_2_, and 5% O_2_. Late-stage *P. falciparum* infected erythrocytes were enriched using a MACS cell separator with LD columns (Miltenyi Biotec #130-042-901) and fluorescently labelled (PKH26 Red Fluorescent Cell Linker Midi Kit (Sigma #MIDI26-1KT) before perfusion in human brain microvessels.

### Microvessel fabrication

Microvessels were prepared by soft lithography and injection moulding of a collagen hydrogel in between polymethylmethacrylate (PMMA) jigs and polydimethylsiloxane (PDMS) stamps, as previously described for human brain microvessels^7^. BBMVEC, BAOEC or HBMVEC were seeded into the collagen at a concentration of 7 × 10^6^ cells/mL Devices were kept for 3 days to allow for vessel forming, replacing medium approximately every 12 h under gravity-driven flow. 1.5 × 10^6^ fluorescently-labelled *T. congolense* parasites and 10 × 10^6 ^*P. falciparum*-infected erythrocytes were perfused at a defined flow rate of 10 μL/min for 15 min. Microvessels were washed with 150 µL of 1X PBS for 10 min, at the same flow rate. Vessels were fixed with 3.7% paraformaldehyde and washed twice with 1X PBS. Parasite binding quantification was performed by confocal microscopy (Zeiss LSM 980 confocal microscope), 10× magnification, with a total scanning with 30-50μm of depth and analysed using ImageJ.

### Immunofluorescence analysis

2D monolayers: Cells were permeabilized using a 2% Bovine Serum Albumin 0.1% Triton-X100 in 1X PBS for 1 h, then perfused with specific primary antibodies (VE-cadherin (Abcam #ab33168), ZO-1 (Thermo Fisher #339194), ß-catenin (Cell Signalling Technology #9587S), or Von Willebrand Factor (Abcam #ab11713)) in permeabilization solution for 1 h at 4 °C, washed with 3 × 20 min with 1X PBS, then stained with 4 µg/mL dihydrochloride (DAPI), phalloidin (Thermofisher #A22287), and/or secondary antibodies (Thermo Fisher #A21244, #A11016, or #A11015) in 2% Bovine Serum Albumin with 5% foetal bovine serum for 1 h at room temperature, and washed 3 × 30 min with 1X PBS.

3D microvessels: The procedure for 3D microvessels was identical to the 2D monolayers with the following exceptions: devices were incubated for 30 min with background buster (American MasterTech #IMI01172E) before permeabilization, primary antibodies were incubated overnight at 4 °C, and 2.5% goat serum was used instead of 5% foetal bovine serum.

Imaging was performed by confocal microscopy (Zeiss LSM 980 confocal microscope), under 10X or 20X magnification and analysed using ImageJ.

### RT-qPCR

cDNA was synthesized from 400 ng of total RNA using the NZY Reverse Transcriptase (NZYtech, Portugal). Next, qPCR was performed using corresponding primers and Power SYBR green PCR master mix (Thermo Fisher # 4367659). Expression results were normalized to the expression of *gapdh* using the 2^−∆Ct^ method. The primer sequences used were as follows: VCAM1 (5’-GAGCTTGGACGTGACCTTCT-3’ and 5’-TGGGTGGAGAATCATCATCA-3’), ICAM1 (5’-GACTTCTTCAGCTCCCCAAG-3’ and 5’-CCCACATGCTATTTGTCCTG-3’), EPCR (5’-GCTACCTGGAGGAGTTCGTG-3’ and 5’-CTTCGAAGAAGACACGGGCT-3’), Claudin-5 (5’-CCTGGACCACAACATCGTGA-3’ and 5’-TCGTACACCTTGCACTGCAT-3’), and GAPDH (5’-GGCGTGAACCACGAGAAGTATAA-3 and 5’-CCCTCCACGATGCCAAAGT-3’). Sequences of VCAM1, Claudin-5, ICAM1, and *gapdh* were previously published^53,54^.

### Detachment assay with flow

Parasites were either isolated from mouse blood by anion exchange chromatography (1/148) (Lanham and Godfrey, 1970)^55^ or harvested from culture (IL3000), stained with 5 mM Vybrant CFDA SE Cell Tracer dye (#V12883, Invitrogen) diluted 1000 times in trypanosome dilution buffer (TDB) (5 mM KCl, 80 mM NaCl, 1 mM MgSO_4_, 20 mM Na_2_HPO_4_, 2 mM NaH_2_PO_4_, 20 mM glucose, pH 7.4), and incubated for 25 min at 34 °C, 5% CO_2_. At the end of the incubation period, parasites were washed and resuspended in TDB, added to the endothelial cell monolayers, and incubated for 1 h at 34 °C, 5% CO_2_. Flow was applied with a perfusion syringe pump containing 1X PBS at defined flow rates, for 1 min each. Parasites were imaged live on a Zeiss LSM 980 (Carl Zeiss Microimaging) with a 20X water- immersion objective (0.8 numerical aperture and 0.55 mm working distance) before and after each flow session. We acquired 10 fields of view per condition (each WSS value), per replicate (3 replicates), with green laser (488 nm, maximum power of 13 mW). For all acquisitions, the software used was ZEN blue edition v.2.6, allowing export of images in czi format.

### Scanning electron microscopy

Microvessels were fixed in Karnovsky solution (2% PFA, 2.5% glutaraldehyde in 0.1 M cacodylate buffer, pH 7.4) at least overnight at 4 °C. First, 200 µl of fixative were added to the inlet and incubated for 15 min. After that, additional 300 µl were added to the device. After fixation, the devices were washed with 0.1 M cacodylate, at 4 °C, opened and the collagen containing the microvessels was removed from the jigs and post-fixed with 2.5% glutaraldehyde in 0.1 M cacodylate buffer pH 7.4, for 1 h at 4 °C. After washing twice with 0.1 M cacodylate, samples were incubated with 2% tannic acid and 4.2% sucrose for 1 h, at 4 °C. Samples were subsequently washed twice with distilled water, stained with 1% methylene blue, embedded in 2% low-melt agarose, and sectioned as 150 µm sections in the vibratome. Sections were kept in water until further processing. Samples were dehydrated in an ascending acetone sequence, critical point dried and sputter coated with platinum (60 s, diffuse coating). Images were acquired with a FEI Quanta 650 FEG scanning electron microscope using the detector LEI for secondary electrons at 10 kV, spot 3, high vacuum and dwell time of 3µs.

### Cytological analysis

To assess cell cycle status of parasites grown without endothelial cells, *T. congolense* IL3000 parasites were grown in MATEK glass-bottom dishes overnight. Non-sequestered parasites were removed by aspiration, fixed with 2% formaldehyde, and airdried on to glass slides for at least 4 h. Then, parasites were rehydrated with 1X PBS, permeabilized with 2% Bovine Serum Albumin - 0.1% Triton- X100 in 1X PBS for 10 min, incubated with 4 µg/ml of 4’,6-diamidino-2-phenylindole (DAPI) in 1X PBS for 10 min and then washed twice for 10 min in 1X PBS. Slides were then mounted with fluoromount-G (Thermo Fisher Scientific) and sealed with a glass coverslip secured with nail polish. Parasites remaining sequestered to the MATEK glass-bottom dish following aspiration were fixed with 2% formaldehyde, permeabilised with 2% Bovine Serum Albumin - 0.1% Triton- X100 in 1X PBS for 10 min, stained with DAPI for 10 min, and washed twice with 1X PBS. Cell cycle status was assessed under a Zeiss LSM980 using a bright field 63X objective and a 561 nm laser.

To assess cell cycle status of parasites sequestered to microvessels, *T. congolense* IL3000 parasites were stained with Hoechst (0.25 µg/mL) and CFDA-SE (1:1000 dilution) for 30 min at 37 °C, after which they were washed to remove traces of the dyes and adjusted to a concentration of 10^7^ parasites/mL in TcBSF1 culture media. Subsequently, 1.5 × 10^6^ parasites were perfused into each bovine aortic microvessel by gravity flow through the inlet. Non-sequestered parasites were collected from the outlet, fixed in 4% formaldehyde and kept at 4 °C until staining. Microvessels containing perfused parasites were incubated for 6 h at 34 °C, 5% CO_2_, after which they were washed by perfusion of 1X PBS at 10 μL/min for 10 min. Vessels were fixed with 3.7% paraformaldehyde and washed twice with 1X PBS. Cell cycle status of parasites sequestered to the microvessels and the non-sequestered ones collected from the outlet was assessed under a Zeiss LSM980 using a bright field 63X objective, 561 nm and 488 nm lasers.

### Intravital imaging

Intravital microscopy involved separate surgeries targeting specific organs, as previously outlined for the brain^56^, lungs and heart, liver, pancreas, spleen, kidneys^57,58^, and adipose tissue. Briefly, mice were anesthetized with a ketamine (120 mg/kg) and xylazine (16 mg/kg) mixture via intra-peritoneal injection. Reflexes were checked, and upon their absence, mice received intravenous injections into the retro-orbital sinus of three markers: Hoechst 33,342 for nucleic acid labelling (stock diluted in dH2O at 100 mg/ml, injection of 40 μg/kg mouse) and 70 kDa FITC-Dextran for intravascular space labelling (stock diluted in 1X PBS at 100 mg/ml, injection of 500 mg/kg mouse). Temporary glass windows (Merk rectangular cover glass, 100 mm × 60 mm) or circular cover glasses (12 mm) of 0.17 mm thickness were implanted in each organ. These windows were secured with stitches or surgical glue. For heart and lung imaging, vacuum immobilization was used to prevent thoracic cavity collapse. Brain imaging utilized semi-closed or open cranial windows, reaching depths of around 190 μm or up to 300 μm into the tissue, respectively.

Imaging sessions were conducted on spinning disc microscopes: Zeiss Cell Observer SD (Carl Zeiss Microimaging, equipped with a Yokogawa CSU-X1 confocal scanner, and an Evolve 512 EMCCD camera and a Hamamatsu ORCA-Flash 4.0 VS camera) or a 3i Marianas SDC (spinning disc confocal) microscope (Intelligent Imaging Innovations, equipped with a Yokogawa CSU-X1 confocal scanner and a Photo-metrics Evolve 512 EMCCD camera). Laser units 405, 488, and 647 were utilized for imaging Hoechst, FITC-Dextran, and AF67-CD31 respectively. Imaging was performed using either an oil-immersion plan apochromat 63X objective with 1.4 Numerical Aperture (NA) and 0.17 mm working distance (WD), or a 40X LD C-Apochromat corrected, water immersion objective with 1.1 NA and 0.62 WD. Images were acquired for 20 s at a rate of 20 frames per second. ZEN blue edition v.2.6., or 3i Slidebook reader v.6.0.22 software was used for all acquisitions.

### NPD-1015 administration and static assays

NPD-1015 resuspended in DMSO at 10 or 20 µM was supplemented to *T. congolense* IL3000 cultures in exponential growth for 24 h. The drug was removed by centrifugation and washing in serum-free media before attachment assays. For attachment assays, 5 × 10^4^ BAOECs per well were seeded on a 96-well plate for 24 h, reaching 80–100% confluency. After this, 2 × 10^5^ parasites were added and incubated for 1 h at 34 °C, 5% CO_2_. Unbound parasites were washed with 1X PBS by vigorous pipetting and remaining cells were fixed with 4% formaldehyde. For detachment assays, 2 × 10^5^ BAOECs were seeded on a 12-well plate and incubated for 24 h at 37 °C, 5% CO_2_. Subsequently, 2 × 10^5^ *T. congolense* IL3000 parasites in TcBSF1 media were added to each well and incubate for 48 h at 34 °C. The supernatant was removed and 1 mL of TcBSF1 media supplemented with 20 µM NPD-1015 or the corresponding volume of DMSO were added to each well and incubated for 24 h at 37 °C, 5% CO_2_. Finally, the supernatant was removed and discarded, whilst remaining cells were washed with 1X PBS and fixed. In both attachment and detachment assays, sequestered parasites were imaged on Nikon HTM-HCS with a 40X long working distance objective and analysed and analysed using ImageJ.

### Cell viability analysis after NPD-1015 administration

T. congolense IL3000 parasites were cultured on a 24-well plate, supplemented with 20 µM NPD-1015 or the corresponding volume of DMSO, and incubated for 24 h at 34 °C, 5% CO_2_. Subsequently, parasite cells were resuspended by vigorous pipetting and 1 µg/mL of propidium iodide (G-Biosciences) was added for dead cell staining. Following an incubation of 15–30 min at 34 °C 5% CO_2_, dead cells were quantified by flow cytometry on the CYTEK Aurora cell analyser. Results were analysed in Floreada.io.

### Differentiation assays

For the differentiation assays, *T. congolense* IL3000 cells were incubated overnight with DMSO or 20 µM NPD-1015 in 5 mL of TcBSF1 media on T25 culture flasks. Non-sequestered parasites were removed from the flasks by pipetting, and remaining sequestered parasites were added additional 5 ml of media. Then, detachment was forced by vortexing for a few seconds. Parasites were centrifuged at 1200xg for 10 min and 3 × 10^6^ parasites per condition were incubated in DTM medium at 27 °C, without CO_2_. The number of live cells were counted at 3 and 5 days after on a haemocytometer. At day 5 post differentiation induction, 10^6^ procyclic cells were passaged into a new T25 flask with 5 mL DTM and counted 24 h after to estimate parasite population growth.

### Tsetse Fly infections

*T. congolense* IL3000 bloodstream forms cryopreserved parasites were cultured in TcBSF1 media on T25 culture flasks, at 34 °C, 5% CO_2_. Twenty-four hours before fly infection, 5 × 10^6^ parasites were supplemented with 20 µM NPD-1015 or the same volume of DMSO. On the experiment day, non-sequestered parasites supplemented with DMSO were collected by pipetting and undisturbed sequestered parasites were washed and collected after vortexing for a few seconds to force detachment. These and drug-treated (non-sequestered) parasites were washed twice by centrifugation at 1200xg for 10 min to remove traces of DMSO and NPD-1015. Parasites were counted with a haemocytometer and fed to experimental teneral (unfed, 0–48 h post-eclosion) male and female tsetse flies (*Glossina morsitans morsitans*) at a concentration of 10^5^ trypanosomes per mL of sterile defibrinated horse blood (TCS Biosciences) via a silicone membrane as previously described^59^. Flies were maintained by feeding on non-infected sterile horse defibrinated blood, and killed by decapitation and midguts were dissected out at days 10 post-infection. Midgut infections were scored after breaking down the entire tissue in a 1X PBS drop, and were classified as heavy, medium or mild, based on the number of parasites observed under the microscope.

### Transcriptomics analysis

Four mice were infected with 2000 *T. congolense* 1/148 parasites and euthanized at days 6 post-infection. Blood was collected by cardiac puncture and mice were perfused with 50 ml heparinized 1X PBS. Brain, gonadal adipose tissue, and kidney were dissected and flash-frozen in liquid nitrogen. Organs were homogenized in Qiazol (Qiagen, UK) with silica beads on a bead beater for 2 rounds of 45 s. RNA was extracted using the RNeasy Universal Plus kit (Qiagen, UK) according to the manufacturer’s protocol and RNA concentration and integrity were checked by fluorometry (Qubit DNA HS, Thermo Fisher Scientific) and parallel capillary electrophoresis (TapeStation, Agilent), respectively. Trypanosome-specific cDNA libraries were prepared using custom primers targeting the spliced leader sequence as previously described^25^, and sequenced as 75 bp single-end reads on the NextSeq 550 platform (Illumina, USA). Reads were aligned to the *T. congolense* IL3000 2018 genome available from tritrypDB version 51 using STAR^60^. The output from read alignment was processed with SAMtools^61^, and transcript abundances were estimated using stringtie^62^. Differential expression between sequestered (blood) and non-sequestered (tissues) samples was performed in R, using edgeR^63^ and limma-voom^64^. Log_2_ Fold change of 1 and *p* value < 0.05 was considered significant. VSG profiling was conducted with VAPPER^30^. Gene set enrichment analysis was conducted with GSEA version 4.3.3.

### Statistics and reproducibility

Sequestration quantification was performed on at least 3 independent microvessels per condition. SEM was performed on one microvessel. RT-qPCR was performed twice, with 2-3 technical replicates each time. NPD-1015 attachment, detachment, and viability assays were replicated 2-4 times. Cytological analysis in vitro and in microvessels were replicated twice. Intravital microscopy data was acquired from 3 to 4 independently-infected mice. In vitro differentiation for procyclic forms was replicated four times. Four mice of the same age were used for targeted transcriptomics. Fly infections were replicated 3 times. Graphs were created with GraphPad Prism (version 10.0.2) or with Rstudio (version 2024.04.2+764). Statistical analyses were performed in GraphPad Prism for ANOVAs, t-tests or mixed-effect models, always correcting for multiple comparisons with either Tukey or Sidak’s tests, or with Rstudio for transcriptomics analyses, using limma-voom and edgeR packages.

### Reporting summary

Further information on research design is available in the Nature Portfolio Reporting Summary linked to this article.

## Supplementary information

Supplementary Material

Description of Additional Supplementary File

Supplementary Data 1

Supplementary Data 2

Supplementary Video 1

Supplementary Video 2

Reporting Summary

## Data Availability

Sequencing reads are available from NCBI under BioProject accession number PRJNA1159173. Source data can be obtained in Supplementary Data 2.
