# Supplementary material for: Bioengineered 3D microvessels and complementary animal models reveal mechanisms of Trypanosoma congolense sequestration
Source: Commun Biol. 2025 Feb 27;8:321. doi: 10.1038/s42003-025-07739-z (PMC11865532; doi:10.1038/s42003-025-07739-z)
Supplement: Supplementary file 1 — Supplementary Material [file 42003_2025_7739_MOESM1_ESM.pdf]

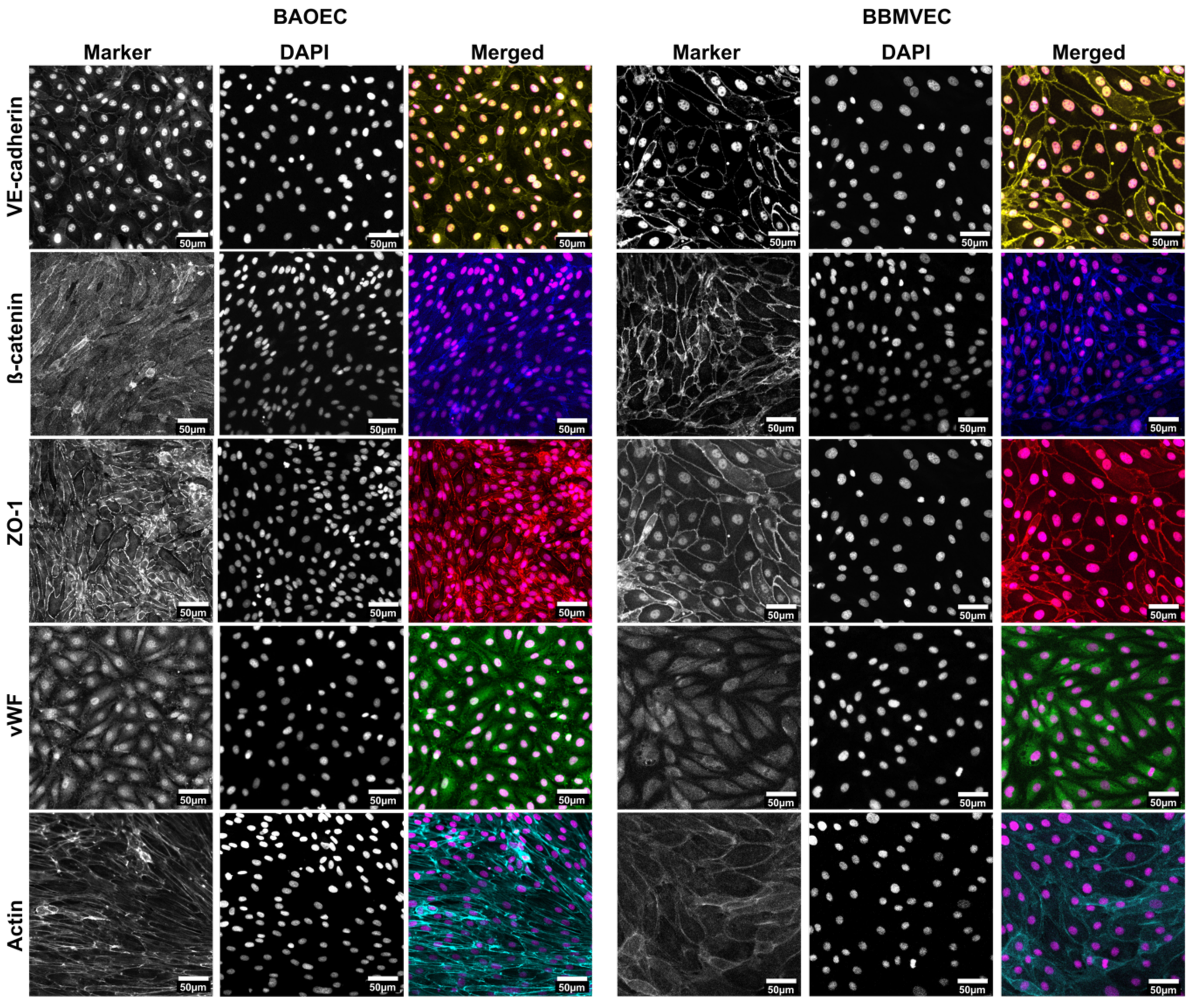

Supplementary Figure 1 Immunofluorescence analysis of bovine aortic endothelial cells and bovine brain endothelial cells grown on a cell monolayer under static conditions. Adherens junction markers (ß-catenin in blue, VE cadherin in yellow), tight junction markers (ZO-1 in red), actin cytoskeleton staining (phalloidin in cyan), pan-endothelial cell marker (Von Willebrand factor in green), and nuclei (4′,6-diamidino-2-phenylindole (DAPI) in magenta). Scale bar = 50µm.

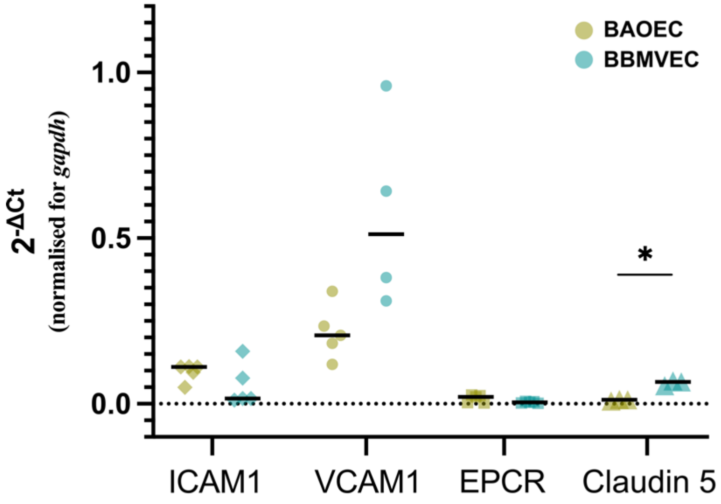

Supplementary Figure 2 Transcript abundance of ICAM1, VCAM1, EPCR and Claudin-5 relative to *gapdh* in bovine aortic endothelial cells (BAOEC) and bovine brain microvascular endothelial cells (BBMVEC). Line indicates the median. Mixed-Effects Model (REML) with Sidak's multiple comparisons test. * indicates p-value < 0.05.

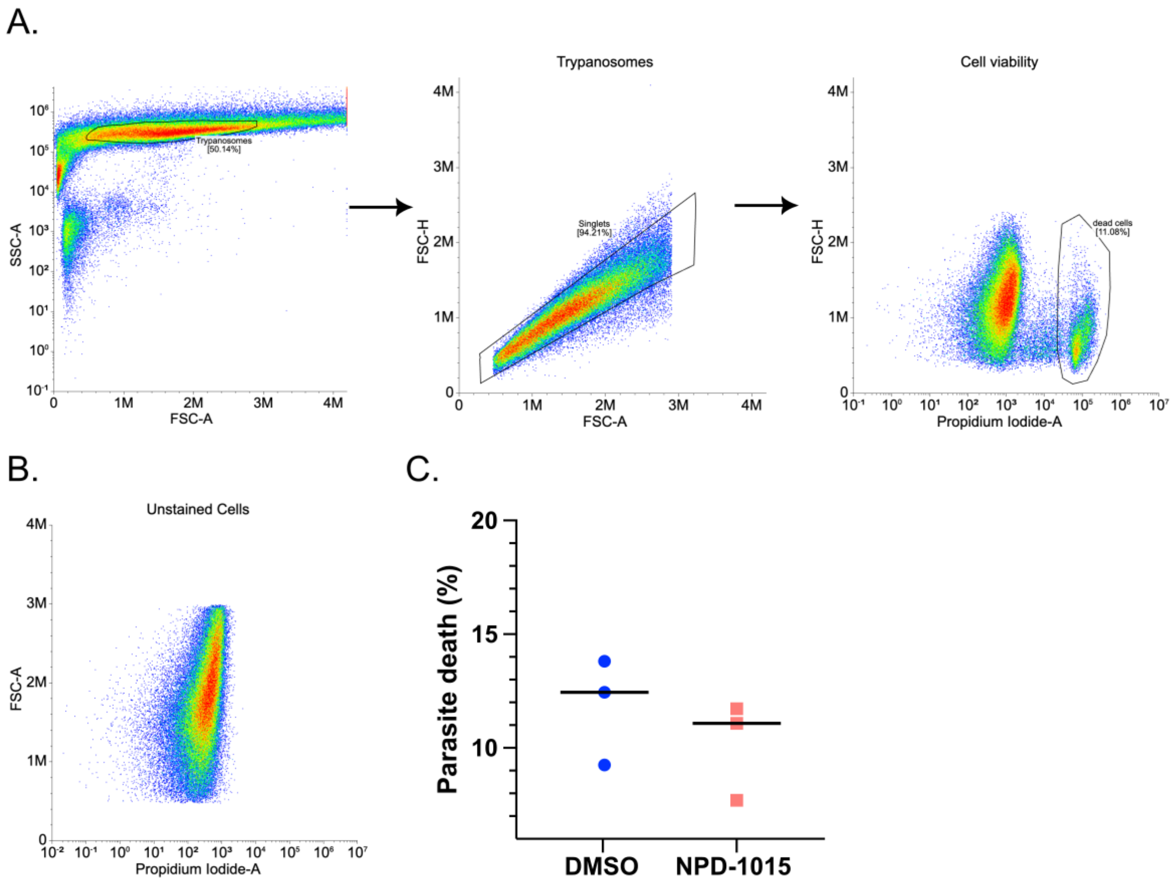

Supplementary Figure 3 Cell viability after treatment with 20μM NPD-1015 or its vehicle (DMSO). A. Gating strategy for the identification of dead trypanosomes: single trypanosomes were selected based on forward (FSC-A) versus side scatter (SSC-A) area; dead trypanosomes were gated as positive for propidium iodide. This strategy was repeated for all samples although only one example is shown. B. Unstained cells gated on propidium iodide are shown as negative control. C. Death of DMSO or NPD-1015-treated parasites calculated as the percentage of propidium iodide-positive cells over the total of single trypanosome cells. Line indicates median.

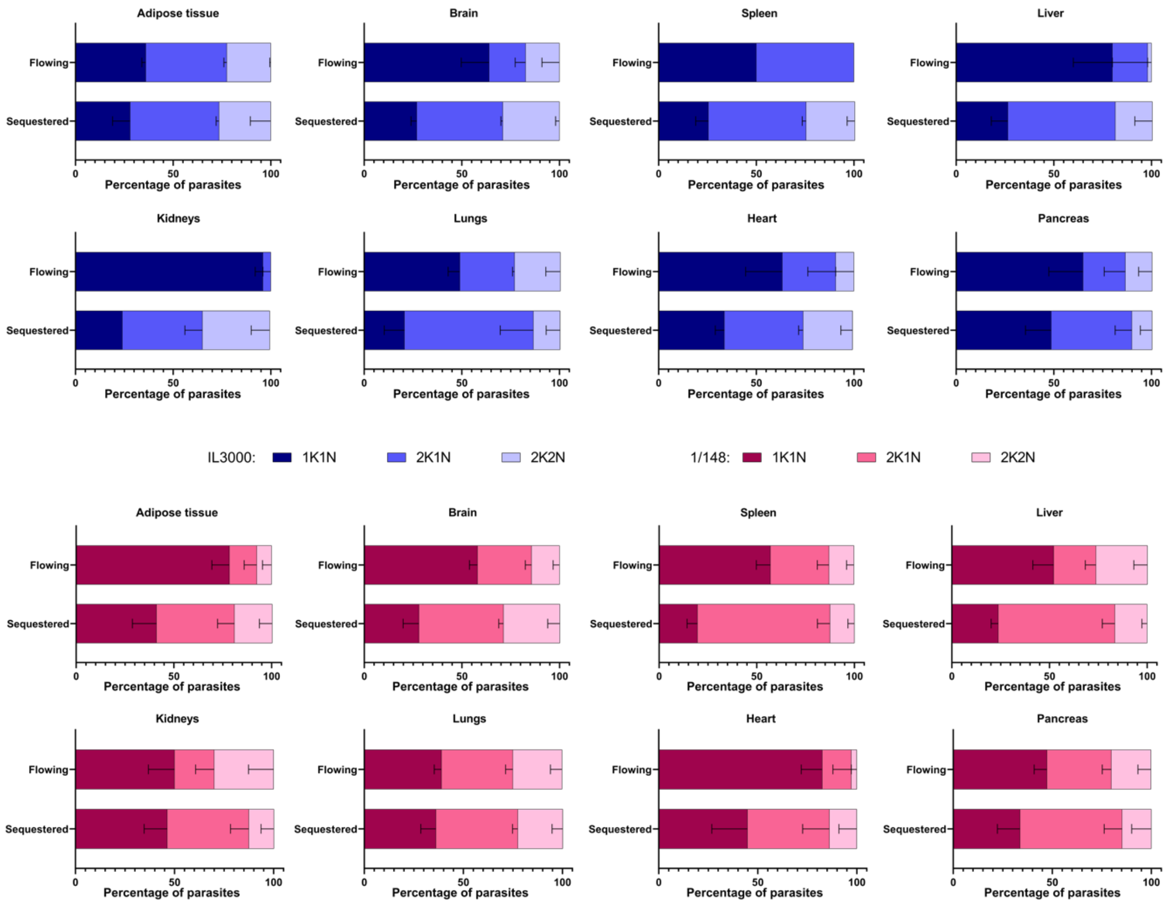

Supplementary Figure 4 Parasite cytological analysis within individual organs of the mouse. Quantification of kinetoplast-nuclei counts of parasites imaged by intravital microscopy. Mice were infected with *T. congolense* IL3000 (blue) or 1/148 (pink), by organ. Nuclei were stained with Hoechst and intravascular environment was stained with 70kDa FITC-dextran. Error bars show standard error of the mean.

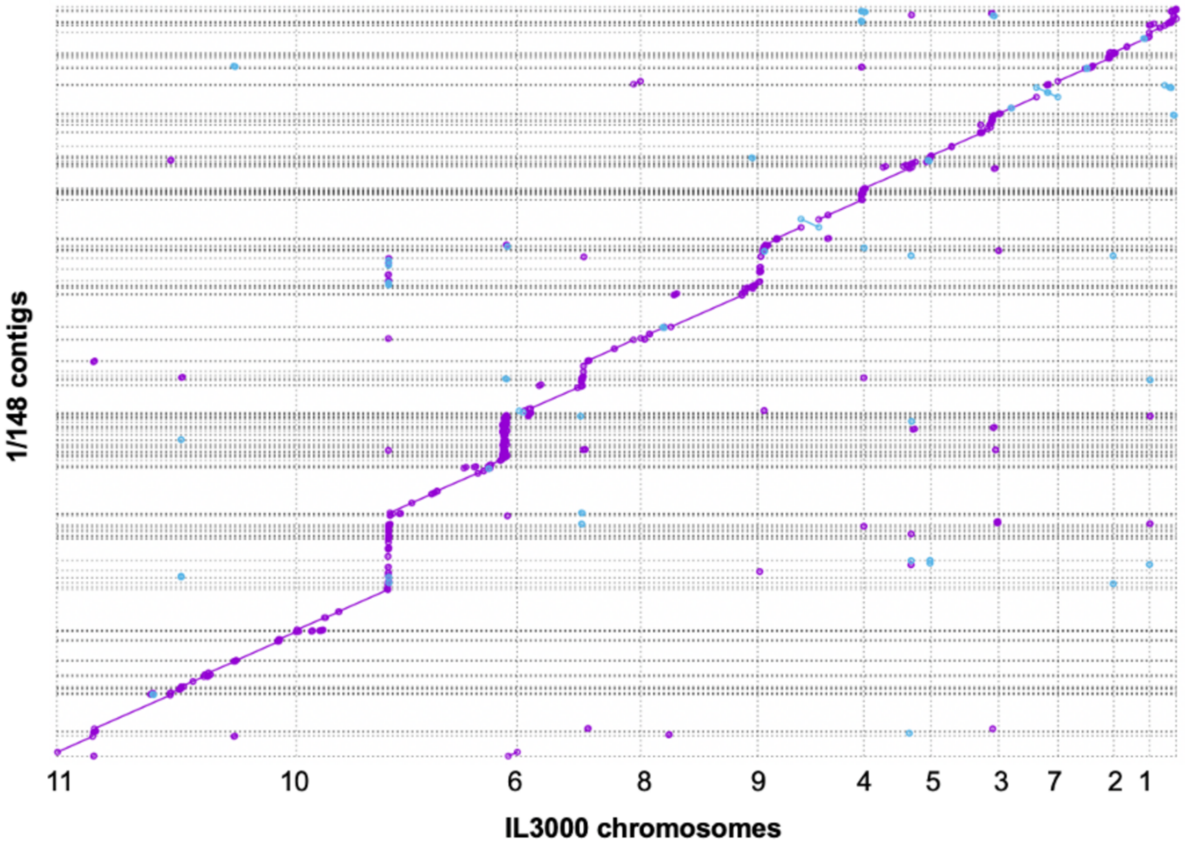

Supplementary Figure 5 Genome homology between *T. congolense* strains IL3000 and 1/148, estimated with mashmap, an approximate aligner for long DNA sequences[66]. Each colour dot/line indicates a match between IL3000 and 1/148. Colours indicate different DNA strands; inverted lines indicate inversions.
