# Supplementary material for: Bioengineered 3D microvessels and complementary animal models reveal mechanisms of Trypanosoma congolense sequestration
Source: Commun Biol. 2025 Feb 27;8:321. doi: 10.1038/s42003-025-07739-z (PMC11865532; doi:10.1038/s42003-025-07739-z)
Supplement: Supplementary file 2 — Description of Additional Supplementary File [file 42003_2025_7739_MOESM2_ESM.pdf]

Description Of Additional Supplementary File

File Name: Supplementary Data 1
Description: Sequencing statistics and differential expression results of sequestered vs. non-sequestered T. congolense parasites.

File Name: Supplementary Data 2
Description: Numerical source data for graphs and charts.

File Name: Supplementary Video 1
Description: Orthogonal view of 3D bovine cardiac microvessel stained with antibodies targeting ß-catenin (red), ZO-1 (green) and DAPI (blue). Scale bar = 30µm.

File Name: Supplementary Video 2
Description: Sequestration of fluorescently-labelled Trypanosoma congolense (in green) to bovine brain microvessels during perfusion, acquired by live imaging. Scale bar = 50µm.
