# Supplementary material for: Bioengineered 3D microvessels and complementary animal models reveal mechanisms of Trypanosoma congolense sequestration
Source: Commun Biol. 2025 Feb 27;8:321. doi: 10.1038/s42003-025-07739-z (PMC11865532; doi:10.1038/s42003-025-07739-z)
Supplement: Supplementary file 7 — Reporting Summary [file 42003_2025_7739_MOESM7_ESM.pdf]

nature portfolio

Corresponding author(s): Luisa Figueiredo, Maria Bernabeu, Sara Silva Pereira, COMMSBIO-24-6533A

Last updated by author(s): 16/01/2025

Reporting Summary

Nature Portfolio wishes to improve the reproducibility of the work that we publish. This form provides structure for consistency and transparency in reporting. For further information on Nature Portfolio policies, see our Editorial Policies and the Editorial Policy Checklist.

Statistics

For all statistical analyses, confirm that the following items are present in the figure legend, table legend, main text, or Methods section.

n/a	Confirmed	
☐	☒	The exact sample size (*n*) for each experimental group/condition, given as a discrete number and unit of measurement
☐	☒	A statement on whether measurements were taken from distinct samples or whether the same sample was measured repeatedly
☐	☒	The statistical test(s) used AND whether they are one- or two-sided<br>*Only common tests should be described solely by name; describe more complex techniques in the Methods section.*
☐	☒	A description of all covariates tested
☐	☒	A description of any assumptions or corrections, such as tests of normality and adjustment for multiple comparisons
☐	☒	A full description of the statistical parameters including central tendency (e.g. means) or other basic estimates (e.g. regression coefficient) AND variation (e.g. standard deviation) or associated estimates of uncertainty (e.g. confidence intervals)
☐	☒	For null hypothesis testing, the test statistic (e.g. *F*, *t*, *r*) with confidence intervals, effect sizes, degrees of freedom and *P* value noted<br>*Give P values as exact values whenever suitable.*
☒	☐	For Bayesian analysis, information on the choice of priors and Markov chain Monte Carlo settings
☒	☐	For hierarchical and complex designs, identification of the appropriate level for tests and full reporting of outcomes
☐	☒	Estimates of effect sizes (e.g. Cohen's *d*, Pearson's *r*), indicating how they were calculated

Our web collection on statistics for biologists contains articles on many of the points above.

Software and code

Policy information about availability of computer code

Data collection	Not applicable
Data analysis	The following open source programs were used: STAR, SAMtools, stringtie, VAPPER, imageJ, Rstudio, Floreada. The following open source R packages were used: EdgeR, limma-voom.<br>The following commercial packages were used: Prism.

For manuscripts utilizing custom algorithms or software that are central to the research but not yet described in published literature, software must be made available to editors and reviewers. We strongly encourage code deposition in a community repository (e.g. GitHub). See the Nature Portfolio guidelines for submitting code & software for further information.

Data

Policy information about availability of data

All manuscripts must include a data availability statement. This statement should provide the following information, where applicable:

- Accession codes, unique identifiers, or web links for publicly available datasets
- A description of any restrictions on data availability
- For clinical datasets or third party data, please ensure that the statement adheres to our policy

Sequencing reads are available from NCBI under BioProject accession number PRJNA1159173.

Research involving human participants, their data, or biological material

Policy information about studies with human participants or human data. See also policy information about sex, gender (identity/presentation), and sexual orientation and race, ethnicity and racism.

Reporting on sex and gender	Not applicable.
Reporting on race, ethnicity, or other socially relevant groupings	Not applicable.
Population characteristics	Not applicable.
Recruitment	Not applicable.
Ethics oversight	Not applicable.

Note that full information on the approval of the study protocol must also be provided in the manuscript.

Field-specific reporting

Please select the one below that is the best fit for your research. If you are not sure, read the appropriate sections before making your selection.

☒ Life sciences ☐ Behavioural & social sciences ☐ Ecological, evolutionary & environmental sciences

For a reference copy of the document with all sections, see nature.com/documents/nr-reporting-summary-flat.pdf

Life sciences study design

All studies must disclose on these points even when the disclosure is negative.

Sample size	Samples sizes of 3-5 were chosen to obtain the minimum required replicate size, whilst still accounting for dropouts.
Data exclusions	No data were excluded from the analysis.
Replication	All presented data has been reproduced, with the exception of the transcriptomic dataset, where 4 independent biological replicates were used instead.
Randomization	Data was not randomized.
Blinding	Investigators were not blinded.

Reporting for specific materials, systems and methods

We require information from authors about some types of materials, experimental systems and methods used in many studies. Here, indicate whether each material, system or method listed is relevant to your study. If you are not sure if a list item applies to your research, read the appropriate section before selecting a response.

Materials & experimental systems

n/a	Involved in the study
☐	☒ Antibodies
☐	☒ Eukaryotic cell lines
☒	☐ Palaeontology and archaeology
☐	☒ Animals and other organisms
☒	☐ Clinical data
☒	☐ Dual use research of concern
☒	☐ Plants

Methods

n/a	Involved in the study
☒	☐ ChIP-seq
☒	☐ Flow cytometry
☒	☐ MRI-based neuroimaging

Antibodies

Antibodies used	Not applicable.
Validation	Not applicable.

Eukaryotic cell lines

Policy information about cell lines and Sex and Gender in Research

Cell line source(s)	Bovine brain microvasculature endothelial cells (BBMVEC) (Cell Applications #B840-05), Bovine aorta endothelial cells (BAOEC) (Cell Applications #B304-05), and Human brain microvascular endothelial cells (HBMEC) (Cell Systems #ACBRI 376) are commercially available. Trypanosoma congolense savannah IL3000 Single marker were kindly provided by Dr Catarina Gadelha at the University of Nottingham. Trypanosoma congolense savannah 1/148 (MBOI/NG/60/1–148) were originally kindly provided by Prof. Alvaro Acosta-Serrano at the Liverpool School of Tropical Medicina.
Authentication	None of the cell lines were authenticated by ourselves.
Mycoplasma contamination	Commercially available cell lines tested negative for mycoplasma contamination.
Commonly misidentified lines (See ICLAC register)	Not applicable.

Animals and other research organisms

Policy information about studies involving animals; ARRIVE guidelines recommended for reporting animal research, and Sex and Gender in Research

Laboratory animals	6-10 weeks old, wild-type, male C57BL/6J mice
Wild animals	Not applicable.
Reporting on sex	Male C57BL/6J mice were used in the study. They were chosen because gonadal adipose tissue was collected for separate analyses and this organ is considerably larger in male compared to female mice, reducing the number of animals required for experimental procedures.
Field-collected samples	Not applicable.
Ethics oversight	This study was conducted in accordance with EU regulations and ethical approval was obtained from the Animal Ethics Committee of Instituto de Medicina Molecular (AWB_2021_11_LF_TrypColonization), the Animal Ethics Committee of the European Molecular Biology Laboratory, and the Animal Ethics Committee of Instituto Gulbenkian de Ciência (A003.2023).

Note that full information on the approval of the study protocol must also be provided in the manuscript.

Plants

Seed stocks	Not applicable.
Novel plant genotypes	Not applicable.
Authentication	Not applicable.
